# Opinion: Substitution Urethroplasty

**DOI:** 10.1590/S1677-5538.IBJU.2015.04.02

**Published:** 2015

**Authors:** Richard A. Santucci

**Affiliations:** 1 Urology, Detroit Medical Center, Detroit, MI, USA; 2 The Center for Urologic Reconstruction™, Detroit, MI, USA; 3 Michigan State College of Medicine, Detroit, MI, USA

**Keywords:** Urethral Stricture, Anastomosis, Surgical, Urethra, Endoscopy

There are many ways to skin a cat. An “index” 2 cm bulbar urethral stricture can be well-treated with an anastomotic technique, with ventral or dorsal buccal urethroplasty, or with direct vision internal urethrotomy. However, urethrotomy has the most limited use. Most experts think direct internal urethrotomy should be reserved for those patients not previously treated, or who are unwilling or unable to have curative urethroplasty. Some published data suggests that patients having their first urethrotomy may have success rates of about 50% (1–3), although we showed much lower success rates of about 8% (4). Very importantly, in one of these series2, the only success was found in patients with very short strictures shorter than 1.5 cm, showing that urethrotomy works poorly in even moderately long strictures In all three studies (1–3), and in our own (4), repeat urethrotomy always failed. The data appears clear: urethrotomy is not an effective treatment for most strictures, and should be expected to fail in nearly all cases where repeat urethrotomy is required.

What of anastomotic vs buccal techniques? I firmly believe that both techniques are valid, but would like to tell you why we (and others: Barbagli, Kulkarni) have largely abandoned the anastomotic approach: COMPLICATIONS. In ours and other expert hands, anastomotic urethroplasty caused a high degree of unacceptable complications. For example, Barbagli reported long-term results from 153 bulbar anastomotic urethroplasties and found a 22% overall sexual complication rate: 14 patients experienced ejaculatory dysfunction, 1 had a cold glans during erection, 7 had soft glans during erections, and 11 had decreased glans sensitivity (5). Morey and Kizer found that 33% of men who had undergone anastomotic urethroplasty for strictures greater than 2.5 cm, suffered decreased penile length after surgery. Curiously, men with shorter strictures less than 2.5 cm in that study had worse outcomes still: 44% had chordee, and 22% had decreased penile length (6). When we directly compared our buccal patients with our anastomotic patients, with long follow-up, anastomotic urethroplasty did poorly in terms of worse success and higher complications. The failure rate of anastomotic urethroplasty was higher than buccal (15% anastomotic vs 8% buccal), even though the buccal patients had in general much longer strictures (1.3 cm anastomotic vs. 3 cm buccal). Anastomotic complications were also higher: 4% chordee and 14% new onset sexual dysfunction, compared to a 0% rate of these problems after buccal urethroplasty. This is in contradistinction to the buccal urethroplasty, which appears to be “exempt of sexual complications…” (7). I'll say that again: exempt of sexual complications.

I believe the things I believe because the data tells me they are so. If you presented me data today that showed me everything I believe is wrong, I would accept it and change my opinion. But the data is clear here, urethrotomy doesn't work very well and anastomotic urethroplasty works well except it has too many complications. Even in the hands of experts (6).
